# Clinical Society of the New York Polyclinic Medical School and Hospital

**Published:** 1913-04

**Authors:** Anthony Bassler


### CLINICAL SOCIETY OF THE NEW YORK POLYCLINIC MEDICAL SCHOOL AND HOSPITAL

(Meeting of April 7th, 1913.)

Cases reported by Dr. Anthony Bassler:

1. Gastric Carcinoma: Operation: Improvement.

This case presented a few unusual features. A man of 70 first seen October 14th, 1912. A moderate drinker. Present illness began with a severe cold after exposure followed by vomiting of a large amount of blood and the general symptoms of hemorrhage. Remained in bed one month. Up to last February lost 50 lbs. Appetite was good but patient did not eat much because of distress after eating, especially after fatty foods. Vomiting was frequent but not constant, longest period elapsing between meal and vomiting was 10 hours. Meat was removed from the diet and since then the bowels have been regular.

Physical examination: Endocarditis, emaciation, stomach resistance, with visible peristalsis. Stomach analysis showed marked hyperacidity, no Boas bacilli or lactic acid. Blood present in stools almost constantly. Placed on a diet of 4,000 colories, consisting of milk and cereals and large amount of butter; the patient gaining one pound per day for 27 days, and gastro symptoms were relieved. The mass in the epigastrium remained the same, X-ray showing it distinctly. Gastroenterostomy was performed: the patient gained 13 pounds in six weeks and then 44 pounds in as many days. The diet is general now and all distressing symptoms have disappeared.

The interesting points are: Carcinoma with hyperacidity, marked improvement after gastroenterostomy and the beneficial use of 10 grains of thymus t. i. d. Three other somewhat similar cases also treated with thymus showed marked improvement.

2. Case of Persistent Brachial Neuritis and Obesity: treated Intestinally: Cure.

This woman 57 years of age was first seen October 2, 1912. Acute indigestion for a number of years with a brachial neuritis. Last summer the painful neuritis extended into arm. Eating of fresh fruits increased the pain. This is the so-called gluteal type of intestinal disturbance. There was pain in the left side of the abdomen. Gain in weight. Physical examination revealed an endocarditis, with occasional dropped beats, otherwise negative. A normal diet was arranged according to the age and weight of the patient and after three days urine showed a great increase in uric acid output and bacterial examination of the feces great numbers of bacillus aerogenous capsulatus. A diet of farinaceous foods, olive oil, etc., and no meat was ordered, vaseline administered to regulate the bowels alkaline drinks and a large dose every fourth day of the Gram negative streptococcus intestinalis capulatus was given to antagonise the aerogenous bacillus. The entire situation now cleared up. After a few weeks the acid output became normal, there was a loss in weight of 40 lbs. and the brachial neuritis disappeared.

Case 3. Case of Arthritis Deformans. Treated intestinally. Cure.

This young woman, a teacher of 34 years old, has had a chronic joint involvement of the hands and feet for the last five years atypical hypertrophic polyarthritis with an occasional acute exacerbation. The stiffness has persisted. During the first attack she was markedly constipated, a psoriasis developed and the patient lost 15 pounds. Placed on a normal diet the urine showed a slight trace of albumen and the toxic substances, uresin, indican and a high oxidizing element. The stools showed abundant bacilli of puerfaction, and a Gram positive diplococci. She was placed on a diet with the smallest amount of calcium salts and bowels regulated by bran and agar. The stools then showed seven distinct strains of Coli communis. Once every four days the patient was given a very large dose of coli bacilli, later placed upon a general diet. Now under treatment for two and a half years and no further attacks of polyarthritis have been experienced.

Case of Cerebella tumor, reported by Dr. R. Foster Kennedy. Operation. Recovery.

A young man 18. Violin player. Immediate history was illness after returning from a party. Continued to have occasional vomiting spells with violent headaches, and transient diplopia, and later inability to manipulate the left little finger. Then these developed unsteadiness in the left leg. The ataxia became so marked that patient was barely able to walk and lurched to the left. Left arm became ataxic. Transient right facial paralysis, very severe headaches, especially in occiput. Examination: Compression pulse, below 60. Orientation good but rather drowsy, nystagmus to right and left, pupils equal and reacted well to light and accommodation. Diminution of sensibility of right fifth nerve, hearing good, trace of weakness in face. Ataxia more marked in left arm and left leg. Both were atonic. Reflexes on left side were increased over those on right side. Abdominal reflexes absent. Right optic disc markedly atrophied and considerable old choroiditis. Left disc showed slight blurring. It was obviously a case of Intra-Pontine cerebellar tumor. While the patient was under observation he collapsed, pulse 40 long sighing respirations; papillitis advanced rapidly. Nystagmus became coarse and irregular to left and absent to right side. Therefore tumor was confined to left cerebella hemisphere. Operation by Dr. Elsberg. Cerebellum was exposed and it immediately protruded by its pressure, the pathological portions were removed. For three weeks the patient had an up and down course with finally complete recovery.

Case 2. Myasthenia gravis. This case presented some points difficult of diagnosis. A young girl of 17 always enjoyed good health. Her present complaint began when she started painting notions in water color. The casual relation however, is not clear. Last October felt eyelid becoming heavy, and had difficulty in keeping it open. In one week it was entirely closed. Left now recovered and right had a similar course. Noticed that she began to see double but the diplopia was not of a constant variety due to the fact that different ocular muscles were affected on different days and therefore the images were variable. On examination there is ptosis of both sides, more marked over the left eye. Compensatory elevation of eyebrows. Complete paralysis for downward movemens of the eyes, movement of right eye laterally is good, of left eye is almost nil. Upward movement of both eyes present. Right pupil larger than the left. Fundi normal. There are no other symptoms except those of the eye. Three possibilities must be thought of in a diagnosis.

First. Inferior polioencephalitis of Mernick. The gradual onset of two weeks and the intactness of the other senses is against this.

Second. Tumor of the Corpora quadrigemini with pressure on the nuclei of the oculomotor nerve. But in six months marked pyramidal and cerebellar signs should have developed.

Third. Myasthenia gravis. It is not unusual in this condition for the symptoms to begin with paralysis of the extrinssic muscles of the eyeball. Againt this is the inequality of the pupils. Myasthenia gravis is a disease of the voluntary muscles of the eyes, and therefore there ought not to be inequality of the pupils. Dr. Pritchard suggested Hysteria as a possible diagnosis. But in that case the symptoms would be limited to ptosis.

---

Severe neuralgic pain over the bridge of the nose isndicates pressure on the anterior ethmoidal nerve, probably due to a high deviation of the nasal septum.—*American Journal of Surgery.*

---

To render a packing introduced for epistaxis easily removable insert a rubber finger cot into the nose, hold it open with clamps and pack the gauze into this.—*American Journal of Surgery.*

